# Node of Ranvier disruption as a cause of neurological diseases

**DOI:** 10.1042/AN20130025

**Published:** 2013-08-07

**Authors:** Keiichiro Susuki

**Affiliations:** Department of Neuroscience, Baylor College of Medicine, Houston, TX, U.S.A.

**Keywords:** axon–glial interactions, ion channel, neurological disease, node of Ranvier, AIDP, acute inflammatory demyelinating polyradiculoneuropathy, AIS, axon initial segment, ALS, amyotrophic lateral sclerosis, AMAN, acute motor axonal neuropathy, Caspr, contactin-associated protein, CIDP, chronic inflammatory demyelinating polyradiculoneuropathy, CMT, Charcot-Marie-Tooth disease, CNS, central nervous system, EAE, experimental allergic encephalomyelitis, EAN, experimental allergic neuritis, ECM, extracellular matrix, GBS, Guillain-Barré syndrome, Kv, voltage-gated K^+^ channels, MS, multiple sclerosis, Nav, voltage-gated Na^+^ channels, NF, neurofascin, PNS, peripheral nervous system, SIMPLE, small integral membrane protein of lysosome/late endosome, Tag1, transiently expressed axonal glycoprotein 1

## Abstract

Dysfunction and/or disruption of nodes of Ranvier are now recognized as key contributors to the pathophysiology of various neurological diseases. One reason is that the excitable nodal axolemma contains a high density of Nav (voltage-gated Na^+^ channels) that are required for the rapid and efficient saltatory conduction of action potentials. Nodal physiology is disturbed by altered function, localization, and expression of voltage-gated ion channels clustered at nodes and juxtaparanodes, and by disrupted axon–glial interactions at paranodes. This paper reviews recent discoveries in molecular/cellular neuroscience, genetics, immunology, and neurology that highlight the critical roles of nodes of Ranvier in health and disease.

## INTRODUCTION

The main function of neurons is to transmit information among various regions of the brain, from the brain to the body, and from the body to the brain. Ideally, information must be transmitted very rapidly over a long distance with minimal energy using the confines available of the nervous system itself. Vertebrates have solved this problem by creating myelinated nerve fibers: structures composed of long neuronal ‘axon’ processes that communicate with targets, and glial cell derived myelin sheaths, multi-lamellar structures that wrap around them. Mostly axons are ensheathed and insulated by serially arranged myelin sheaths along their entire length. These structures are formed through the interaction between neurons and myelin-forming glial cells, Schwann cells in PNS (peripheral nervous system), and oligodendrocytes in CNS (central nervous system). Myelination is initiated by signals from axons to the glial cells (Sherman and Brophy, 2005; Pereira et al., 2012). In turn, myelinating glial cells actively promote formation of distinct membrane domains on the axons including the nodes of Ranvier, short gaps between two adjacent myelin segments or internodes (Poliak and Peles, 2003; Salzer, 2003; Rasband, 2011). Voltage-gated ion channels, cell adhesion molecules, and cytoskeletal and scaffolding proteins characterize nodal regions. Keys among these molecules are the Nav (voltage-gated Na^+^) channels that regenerate action potentials. Since the axons between nodes are insulated by myelin sheaths, the action potentials are regenerated only at the nodes allowing rapid and efficient propagation in a saltatory manner.

Recent progresses in molecular and cellular biology have revealed the mechanisms that control the assembly of the nodes of Ranvier. Furthermore, increasing knowledge on basic neurobiology also has been providing new understanding of the mechanisms underlying nervous system diseases or injuries. Since nodal Nav channel clusters are critical for action potential transmission, it is not surprising that changes in Nav channels induce neurological symptoms. Furthermore, since the formation and maintenance of nodes depend on neuron–glia interactions, both glial cell/myelin defects and axonal damage can disrupt nodal Nav channel clusters and consequently cause nerve conduction failure. Traditionally, mechanisms of diseases involving myelinated nerve fibers are categorized as either demyelinating or axonal. However, I propose that dysfunction or disruption of nodes of Ranvier should also be a focus for understanding the pathophysiology of neurological diseases. This paper reviews the recent evidence for a pathogenic role of nodal dysfunction and/or disruption during neurological diseases.

## MOLECULAR ORGANIZATION AT AND NEAR THE NODES OF RANVIER

**Figure 1:**
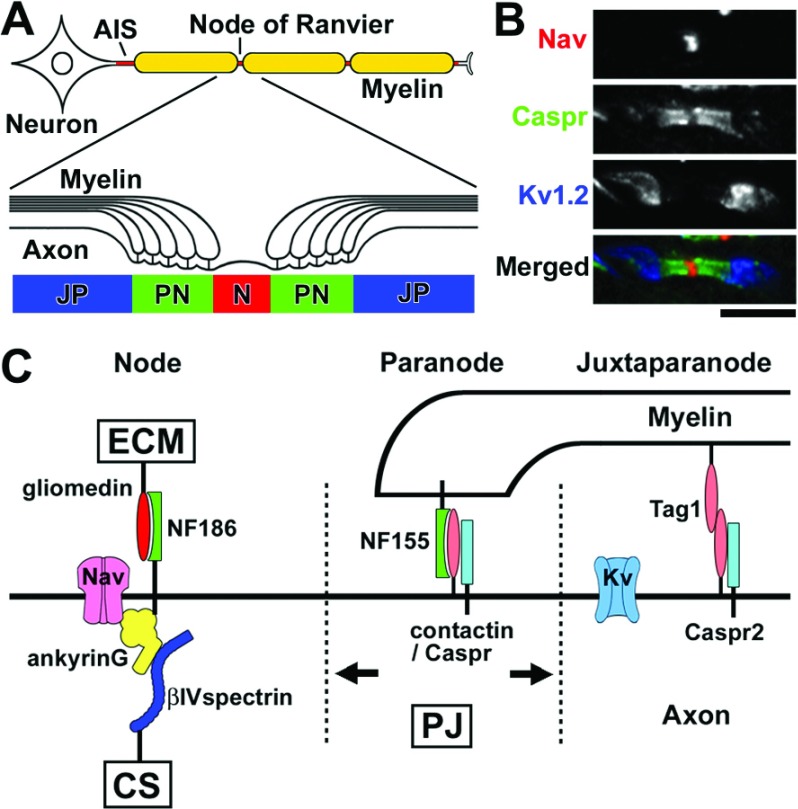
Molecular composition at nodes of Ranvier (**A**) Cartoon illustrating the structures of the myelinated nerve fiber and axonal subdomains: AIS, nodes of Ranvier (N), paranode (PN), and juxtaparanodes (JP). (**B**) Longitudinal sections of mouse optic nerve section immunostained with antibodies to Nav channels (nodal marker, red), Caspr (paranodal marker, green), and Kv1.2 channels (juxtaparanodal marker, blue). (**C**) Schematic presentation showing molecular organization at nodes, paranodes, and juxtaparanodes, and proposed three complementary mechanisms for node assembly. Gliomedin is the ECM component in the PNS that interacts with NF186. Paranodal junctions (PJ) restrict the mobility of membrane molecular complexes at nodes or juxtaparanodes. Cytoskeletal scaffolds (CS) further stabilize the Nav channel complex at nodes.

Neurons are highly polarized cells and the myelinated axons are divided into multiple distinct membrane domains including AIS (axon initial segments), nodes of Ranvier, paranodes, juxtaparanodes, internodes, and terminals (Figure 1A). Specific molecular complexes characterize each of these domains (Figures 1B and 1C) [for more detail, see (Poliak and Peles, 2003; Salzer, 2003; Rasband, 2011)]. The AIS share a common molecular organization with nodes of Ranvier (Rasband, 2010). The Nav channels accumulate at the AIS to initiate the action potentials, whereas at the nodes they are responsible for the action potential propagation. The major Nav channel subtype in mature nodes is Nav1.6 in both CNS and PNS. During nodal development, Nav1.2 channels are present first, but are then mostly replaced by Nav1.6. In addition, Nav1.1 is also found at adult CNS nodes and AIS (Duflocq et al., 2008). Nav channels at nodes and AIS are multimers, and may include a single α-subunit associated with one non-covalently (β1 or β3) and one covalently (β2 or β4) linked β-subunit. Nav channel β-subunits have been proposed to modulate Na^+^ current and function as cell adhesion molecules [reviewed in (Patino and Isom, 2010)]. In addition to Nav channels, Kv (voltage-gated K^+^) channels, KCNQ2, and KCNQ3, are clustered in higher densities at nodes and the AIS where they modulate axonal excitability. KCNQ2 channels mediate the slow nodal K^+^ current regulating the excitability of nodal axons (Schwarz et al., 2006). At the nodal axolemma, the scaffolding protein ankyrinG binds with Nav channels, the cell adhesion molecule NF (neurofascin) 186, and the cytoskeletal protein βIV spectrin to form a large complex. At the flanking paranodes, axonal contactin and Caspr (contactin-associated protein) and glial NF155 form a tripartite cell adhesion molecule complex that mediates the assembly of the septate-like junctions between the myelin sheath and the axon. Three mechanisms operate conjointly during nodal assembly: (1) clustering of NF186 by a glia-derived ECM (extracellular matrix), (2) restriction of nodal membrane protein mobility by paranodal junctions, and (3) stabilization of Nav channels by axonal cytoskeletal scaffolds (Figure 1C) (Susuki and Rasband, 2008). There are some differences in the molecular organization and assembly mechanisms between PNS and CNS nodes. ECM molecules that cluster NF186 are: gliomedin and NrCAM in the PNS (Eshed et al., 2005; Feinberg et al., 2010); and brevican, versican V2, Bral1, and NrCAM in the CNS (Susuki et al., 2013). Among the three mechanisms described above, the ECM is the primary one in the PNS (Feinberg et al., 2010; Susuki et al., 2013), whereas paranodes drive nodal assembly in the CNS (Susuki et al., 2013). Juxtaparanodes flank paranodes and reside under the myelin sheaths (Figure 1C). Juxtaparanodal molecules include Kv1 channels, Caspr2, and Tag1 (transiently expressed axonal glycoprotein 1). Paranodal junctions also restrict the mobility of these juxtaparanodal proteins. Juxtaparanodal Kv channels are thought to act as an active damper of re-entrant excitation to help in restoring and maintaining the internodal resting potential, to provide a protective function in axons that might undergo a low degree of demyelination, and to mediate axon–glial communication [reviewed in (Poliak and Peles, 2003)].

## NODAL DYSFUNCTION/DISRUPTION IN NEUROLOGICAL DISEASES

### Dysfunction of ion channels at and near nodes

As action potential propagation is regulated by the activity of Nav channels highly clustered at the nodes of Ranvier, changes in Nav channel kinetics are likely to profoundly impact on nerve transmission. A clear example where the dysfunction of nodal Nav channels causes nerve conduction failure is tetrodotoxin poisoning, the result of consuming puffer fish (Isbister and Kiernan, 2005). Tetrodotoxin blocks Na^+^ conductance by binding extracellularly at receptor-site 1 of Nav channels preventing monovalent cations from accessing the pore. In severe cases, patients rapidly develop generalized flaccid paralysis that can progress to respiratory failure and even death. Electrophysiological examinations of peripheral nerves of patients with puffer fish poisoning show reduction of compound action potentials in both motor and sensory nerves, and altered nerve excitability properties consistent with tetrodotoxin blockade of Nav channel pores (Kiernan et al., 2005). Reduced nerve excitability because of Nav channel inactivation underlies weakness in critically ill patients (Novak et al., 2009). Several disorders of neuronal excitability such as epilepsy are linked to mutations in Nav channels or alterations in Nav channel trafficking [reviewed in (Eijkelkamp et al., 2012)]. For example, mutations in *SCN8A* encoding Nav1.6, a major Nav channel subtype expressed at mature nodes of Ranvier, are associated with mental retardation, ataxia, and cerebellar atrophy (Trudeau et al., 2006). Recently, a *de novo* heterozygous missense mutation in *SCN8A* was identified in a patient with a severe epileptic encephalopathy consisting of early onset seizures, features of autism, intellectual disability, ataxia, and sudden unexplained death in epilepsy (Veeramah et al., 2012). Furthermore, Dravet syndrome, one of the most severe forms of childhood epilepsy, is caused by mutations in *SCN1A* encoding Nav1.1 [reviewed in (Eijkelkamp et al., 2012)], or a mutation in *SCN1B* encoding Nav channel β1 subunit (Patino et al., 2009). Similarly, KCNQ2 and KCNQ3, enriched at the nodes and AIS, are mutated in patients with an autosomal dominant epilepsy syndrome called benign familial neonatal convulsions [reviewed in (Cooper, 2012)]. It is not clear if ion channel dysfunctions specifically at the nodes alone underlie the development of these diseases, since there are also high densities of these ion channels at the AIS, and low densities in somatodendritic regions and in internodal axons. However, some evidences suggest a role of ion channel dysfunction at or near nodes on the development of neurological symptoms. For example, a mutation in KCNQ2 may cause myokymia (Dedek et al., 2001), involuntary contractions of skeletal muscles indicative of hyperexcitability in myelinated motor axons, presumably because of altered slow nodal K^+^ current. Kv1.1 channels located at juxtaparanodes have a profound stabilizing effect on the action potential when it reaches the transition zone near the nerve terminal (Zhou et al., 1999), and mutations in *KCNA1* encoding Kv1.1 cause episodic ataxia and myokymia [reviewed in (Jen et al., 2007)].

These findings emphasize the importance of properly functioning ion channel clusters at and near the nodes of Ranvier. Furthermore, it is easy to speculate that, in the neurological diseases involving myelinated nerve fibers, altered functions of nodal Nav channels and juxtaparanodal Kv channels lead to conduction failure. Indeed, the disruption of the molecular organization, altered ion channel expression, function, location, and/or density at the AIS are emerging as key players in the pathophysiology of neurological disorders [reviewed in (Buffington and Rasband, 2011)]. Axonal injury, demyelination, or both can disrupt nodes of Ranvier and changes in their functions may contribute to the pathophysiology of various neurological diseases as described below.

### Autoimmune reactions targeting nodes of Ranvier

In some immune-mediated neurological diseases, the autoimmune processes specifically target molecules concentrated at nodes of Ranvier. The best example is the autoimmune neuropathies called GBS (Guillain-Barré syndrome) characterized by acute progressive limb weakness. GBS is divided into two subtypes, an axonal form [AMAN (acute motor axonal neuropathy)] and a demyelinating form [AIDP (acute inflammatory demyelinating polyradiculoneuropathy)] (Yuki and Hartung, 2012). Most patients with AMAN have serum IgG antibodies against gangliosides, a group of acidic glycosphingolipids with single (e.g. GM1) or multiple (e.g. GD1a and GD1b) sialic acids. These gangliosides, abundantly expressed on neuronal cell membrane, are highly enriched at and near nodes, and have various neurobiological functions that may include maintenance of the axon, myelin integrity, and/or stabilization of axon–glial interactions (Sheikh et al., 1999; Yamashita et al., 2005; Susuki et al., 2007a). In human AMAN, an early pathological feature is widening of the nodes of Ranvier with no or little demyelination in ventral roots (Griffin et al., 1996). The affected nodal axolemma is coated with activation products of complement, key components of the innate immune systems (Hafer-Macko et al., 1996a). Complement-derived chemotropic signals may recruit macrophages to the affected nodes. A characteristic nerve conduction study finding in AMAN patients is the rapidly reversible conduction failure with no signs indicating remyelination (Kuwabara et al., 1998; Kokubun et al., 2010). As this type of conduction failure cannot be explained by the recovery from demyelination or axonal degeneration, dysfunction of Nav channels at nodes is a likely underlying cause. Thus, it has been suggested that the anti-ganglioside antibodies bind to the nodal axolemma, activate the complement pathways that disrupt the nodal structure and its molecular organization. The initial nodal disruption may be repaired rapidly. If the local immune reaction progresses, then axons will degenerate. For further information on the clinical courses and outcomes of AMAN, see (Uncini et al., 2013).

**Figure 2:**
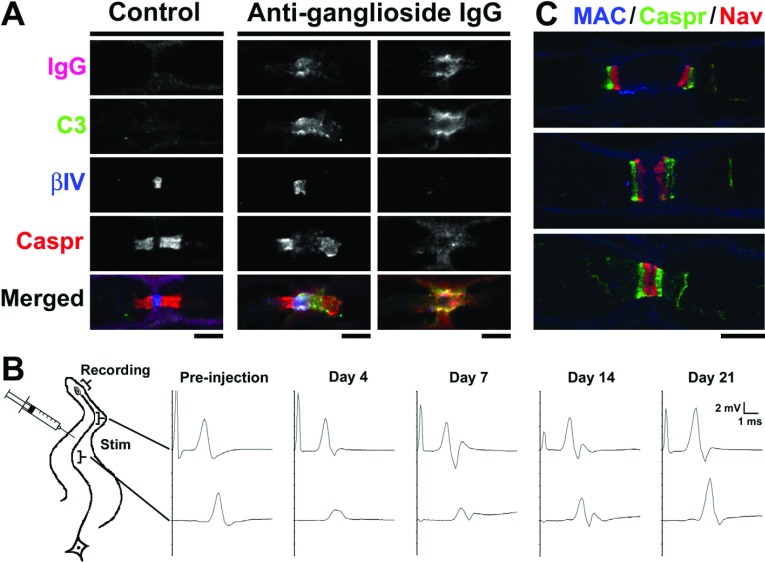
Immune-mediated attack selectively targeting nodes of Ranvier (**A**) Immunofluorescence analyses in longitudinal sections of rat sciatic nerves 4 days after injection of control IgG or mouse monoclonal IgG to gangliosides GD1a and GT1b. In control, no IgG (magenta) or C3 component of complement (green) is detected at node, and nodal βIV spectrin (blue) and paranodal Caspr (red) are normally distributed (left column). Depositions of anti-ganglioside antibodies and complement are associated with the abnormally lengthened gap between paranodal Caspr clusters with preserved nodal βIV spectrin (middle column), or with completely damaged βIV spectrin and Caspr clusters (right column). (**B**) Serial nerve conduction study in rat tibial nerve. IgG anti-ganglioside antibody was injected half way between ankle and knee. The nerve is stimulated at ankle or knee, and the compound muscle action potentials are recorded from plantar muscle. Before injection, no apparent difference is seen between the waveforms after stimulation at the ankle and knee. After anti-ganglioside IgG injection, the amplitude after stimulation at the knee is abnormally reduced with no temporal dispersion, suggesting the presence of nerve conduction block in the region of anti-ganglioside antibody injection. The amplitude after proximal stimulation returned to normal by 21 days after injection. (**C**) Ventral root from rabbit inoculated with GM1 ganglioside during early recovery phase (2 weeks after the onset of neurological disease). Binary Nav channel clusters associated with Caspr on both sides of affected nodes (top panel). The intensity of MAC staining is reduced. Two adjacent Nav channel clusters are present very close (middle panel) or appear to fuse (bottom panel). (**A**) and (**C**) are reprinted with permission from J Neurosci 27(15) 3956–3967, Susuki K, Rasband MN, Tohyama K, Koibuchi K, Okamoto S, Funakoshi K, Hirata K, Baba H, Yuki N. Anti-GM1 antibodies cause complement-mediated disruption of sodium channel clusters in peripheral motor nerve fibers (2007). With permission from the Society for Neuroscience. (**B**) is reprinted from Experimental Neurology, 233(1), Keiichiro Susuki, Nobuhiro Yuki, Dorothy P. Schafer, Koichi Hirata, Gang Zhang, Kei Funakoshi, Matthew N. Rasband, Dysfunction of nodes of Ranvier: A mechanism for anti-ganglioside antibody-mediated neuropathies, 534–542, Copyright (2012), with permission from Elsevier.

Several animal studies support this notion. In ventral roots from a rabbit model of AMAN induced by immunization with gangliosides, nodes were abnormally lengthened, and Nav channel immunostaining was lost as IgG and complement accumulated (Susuki et al., 2007b). Nodal and paranodal molecules are disrupted in association with complement deposition but without direct contact by macrophages. Macrophage infiltration was most prominent during the early recovery phase, suggesting that they play a role in the clearance of degenerated nerve fibers. Furthermore, passive transfer of anti-ganglioside antibodies into rodent peripheral nerves reproduced the complement-mediated disruption of nodal Nav channel clusters resulting in nerve conduction block (Figures 2A and 2B) (McGonigal et al., 2010; Susuki et al., 2012). Disruption of the nodal and paranodal molecular complex is often mediated by calpain, a calcium-dependent protease, an enzyme activated with accelerated calcium influx through membrane pores formed by insertion of MAC [(membrane attack complex)–a final product of the complement pathway] in the nodal axolemma (McGonigal et al., 2010). Nodal molecules including Nav channels, ankyrinG, or βIV spectrin, and neurofilament in the axonal cytoskeleton are targets of proteolysis by calpain, and their breakdown might cause nodal disruption and subsequent axon degeneration. During the recovery phase, characteristic of AMAN, complement deposition was reduced, and nodal and paranodal molecules again cluster on both sides of the affected nodes (Figure 2C) (Susuki et al., 2007b). Binary Nav channel clusters then appear to fuse allowing the reformation of nodes of Ranvier. Similar to the reversible conduction failure in AMAN patients, the nerve conduction block induced by injecting anti-ganglioside IgG into rat sciatic nerve was rapidly resolved (Figure 2B) (Susuki et al., 2012). This type of nerve conduction block may involve multiple factors including: (1) nodal ionic imbalance due to the bi-directional, non-specific ion and water pores formed by the insertion of MAC into the nodal axolemma (McGonigal et al., 2010), (2) reduced numbers of functioning Nav channels, (3) large leakage of driving current resulting from paranodal detachment, and (4) exposure of juxtaparanodal Kv1 channels to the nodal area. Indeed, one study supported the above possibilities by showing that, in a single rat myelinated nerve fiber preparation, anti-GM1 antibodies decrease the Na^+^ current and cause a progressive increase of non-specific leakage current in the presence of active complement (Takigawa et al., 1995). However, another study failed to confirm these results (Hirota et al., 1997).

Similar to the situation with AMAN, serum IgM antibodies to GM1 ganglioside are frequently detected in multifocal motor neuropathy, a rare inflammatory neuropathy characterized by slowly progressive, asymmetric distal limb weakness with no sensory loss (Vlam et al., 2011). IgM anti-GM1 antibodies from patients bound to the nodes of Ranvier in rodent sciatic nerves, but did not induce conduction failure (Harvey et al., 1995; Paparounas et al., 1999). Another study reported induction of nerve conduction block by intraneural injection of patient sera into rat tibial nerves (Uncini et al., 1993). Based on the similarity to AMAN, pathophysiology of autoimmune reactions is speculated to cause nodal disruption, although this is not proven due to lack of human pathology or an experimental model. In addition, both animal models of the active immunization of GD1b ganglioside and intraneural injection of IgG anti-GD1b antibody induce complement-mediated nodal disruption predominantly in sensory fibers, suggesting the underlying pathophysiology in human acute sensory ataxic neuropathy associated with anti-GD1b antibodies (Susuki et al., 2012). These findings demonstrate that the autoimmune lesions at nodes of Ranvier disturb nerve conduction, and cause neurological diseases.

### Autoimmunity against nodal, paranodal, and juxtaparanodal proteins

In addition to gangliosides, some studies have identified autoantibodies against nodal, paranodal, or juxtaparanodal proteins in sera from patients with autoimmune neurological diseases. A small portion of patients with GBS or CIDP (chronic inflammatory demyelinating polyradiculoneuropathy) had autoantibodies to gliomedin, NF186, NF155, contactin, or contactin/Caspr complex (for protein localization, see Figure 1C) (Prüss et al., 2011; Devaux et al., 2012; Ng et al., 2012; Querol et al., 2013). The pathogenic roles of these autoantibodies in human patients have yet to be tested (Hughes and Willison, 2012). However, animal studies suggest that these autoantibodies could contribute to the severity of AIDP and CIDP. In EAN (experimental allergic neuritis) induced in the Lewis rat by immunization against peripheral myelin, NF186 and gliomedin were often undetectable prior to demyelination, and autoantibodies to these molecules were found (Lonigro and Devaux, 2009). Furthermore, immunization against gliomedin induced a progressive neuropathy in Lewis rats characterized by conduction defects and nodal disruption in spinal nerve roots (Devaux, 2012). Passive administration of anti-gliomedin IgG into the EAN model (induced by immunization against the neuritogenic P2 peptide) augmented demyelination and nodal disruption, and exacerbated the disease. Similarly, the administration of two different monoclonal antibodies against pan-NF into the EAN model exacerbated the disease (Ng et al., 2012).

Antibodies to NF186 or NF155 were also detected in patients with MS (multiple sclerosis), an autoimmune disease of the CNS myelin (Mathey et al., 2007). Monoclonal antibody to pan-NF administered to the animals with EAE (experimental allergic encephalomyelitis), an animal model for MS, bound selectively at nodes of Ranvier in spinal cords together with complement, induced acute axonal injury, and exacerbated the clinical disease (Mathey et al., 2007). In addition, antibodies to βIV spectrin, a submembranous cytoskeletal protein located in AIS and in nodes of Ranvier (Figure 1C), were detected in a patient with paraneoplastic lower motor neuron syndrome associated with breast cancer (Berghs et al., 2001). After the removal of the cancer, the titer of the autoantibodies and binding at AIS drastically reduced, and neurological symptoms partially improved, suggesting an autoimmune mechanism for the disease. The primary lesion site(s) remains unknown; AIS, nodes of Ranvier, or both. These autoantibodies against nodal and paranodal proteins may cause Nav channel dysfunction similar to the situation in AMAN. Furthermore, since these molecules are involved in the formation and stabilization of Nav channel clusters at nodes (see Figure 1C), their disruption may adversely impact node assembly and/or maintenance; consequently destabilizing nodal Nav channels and thereby damaging neural transmission.

The molecules that accumulate selectively at juxtaparanodes are also potential autoimmune targets. Antibodies to the molecular complex including Kv channels (sometimes termed VGKC-complex antibodies in the literature) are detected in patients with Morvan syndrome, a rare complex disease that combines neuromyotonia with multi-organ autonomic disturbance, insomnia, and encephalopathy (Irani et al., 2012). Target molecules of the Kv channel complex include the juxtaparanodal proteins Caspr2 and Tag1 (also known as contactin-2). Human sera containing Caspr2 antibodies bind to juxtaparanodes in PNS (Irani et al., 2010; Lancaster et al., 2011). Thus, it has been suggested that binding of Caspr2 antibodies may down-regulate Caspr2/Kv1 channel complexes in the PNS axon, leading to neuromyotonia or neuropathic pain due to altered Kv1 channel function. Autoimmunity mediated by autoantibodies and T-cells against Tag1 was also reported in human MS (Derfuss et al., 2009). Pathogenic effects of Tag1-specific T-cells were demonstrated in EAE, an animal model of MS. Taken together, these findings suggest that the functional domains at and near nodes are potential targets of autoimmune reactions, and, consequently, immune-mediated nodal disruption may underlie the pathophysiology of a broad range of neurological diseases.

### Nodal disruption caused by demyelination in the PNS

**Figure 3:**
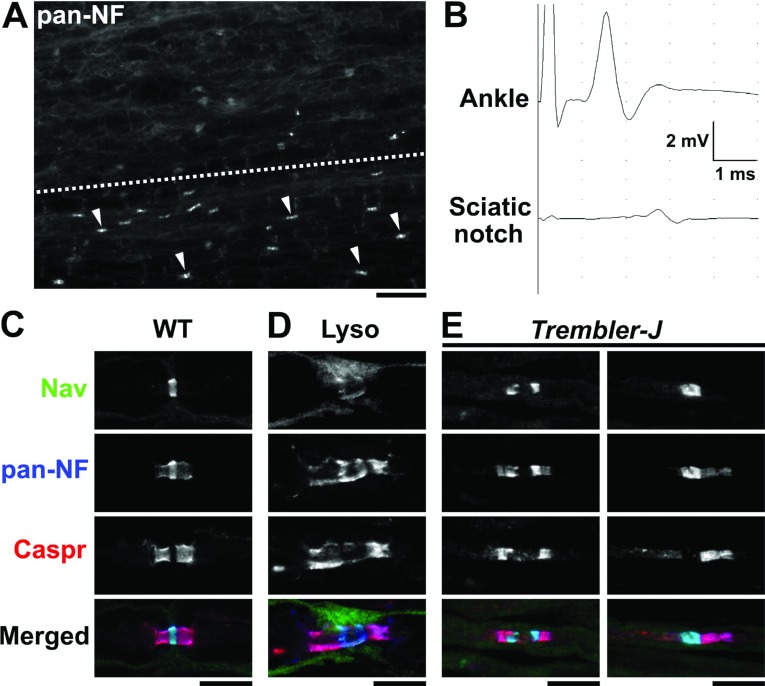
Altered localization of nodal and paranodal components in demyelination models (**A**) Longitudinal section of mouse sciatic nerve seven days after injection of lysolecithin. In the preserved area (below dotted line), immunostaining by anti-pan-NF antibodies shows clusters of strong nodal signals associated with relatively weak paranodal staining (some of them are indicated by arrow heads). In the demyelination lesion (above the dotted line), NF clusters are highly reduced in numbers. Scale bar = 40 μm. (**B**) Nerve conduction study along mouse sciatic nerve seven days after injection of lysolecithin. The nerve is stimulated at distal (ankle) or proximal (sciatic notch) to the injection site, and the compound muscle action potentials are recorded from plantar muscle. The amplitude of compound muscle action potential recorded after stimulation at sciatic notch is highly reduced compared with the ankle indicating the nerve conduction failure. (**C**–**E**) Longitudinal sections of mouse sciatic nerves are immunostained as indicated. Nerve fibers run horizontally. Scale bars = 10 μm. (**C**) WT (Wild-type). Note the anti-pan-NF antibodies display both nodal NF186 (strong signal colocalized with Nav channel staining) and paranodal NF155 (relatively weak signal colocalized with Caspr staining). (**D**) Demyelination model induced by intraneural injection of lysolecithin (seven days after injection). Nodal cluster of Nav channel and NF186 is remarkably dispersed. (**E**) *Trembler-J* heterozygote mouse. Left panel shows markedly elongated nodal gap and binary cluster of Nav channels. Right panel shows Nav channel cluster at heminode: paranodal molecules are only clustered at the right side of the node.

Given the role of myelinating glial cells in organizing and maintaining nodes, it is not surprising that myelin defects disrupt ion channel clustering at and near nodes. Indeed, the altered nodal, paranodal, and juxtaparanodal molecular organization due to demyelination has been clearly demonstrated in animal models such as lysolecithin-induced demyelination (Figures 3A–3D) (Dugandzija-Novaković et al., 1995: Arroyo et al., 2004). Demyelination can affect ion channel clusters in at least two ways. It can: (1) alter the localization of ion channels, and (2) cause abnormal expression of ion channel subtypes, as reported in pathological studies of inherited demyelinating neuropathies and in animal models.

In patients with hereditary neuropathies CMT (Charcot-Marie-Tooth disease), skin biopsy showed that Caspr immunostaining was spread from paranodes into both juxtaparanodes and internodes and Kv channels were distributed in patches along the internodal axolemma rather than remaining in juxtaparanodes (Li et al., 2005). These changes were not observed in a second study (Saporta et al., 2009). Biopsy of sural nerve from a patient with CMT-type 1B, caused by mutations in the myelin protein zero gene, showed that Caspr was no longer localized to paranodes but was spread into demyelinated regions of sural nerve axons (Bai et al., 2006). Structural abnormalities of the node may underlie the changes in the excitability in motor axons of patients with hereditary neuropathy with liability to pressure palsies (Jankelowitz and Burke, 2013). In addition, remodeling of the nodal ECM occurs in CMT type 1 pathology: tenascin, normally accumulated at the nodal ECM, was displaced and extended along the internodes in sural nerves from patients with CMT type 1 (Palumbo et al., 2002). These changes in the nodal ECM may affect nerve conduction, because the ECM, including tenascin, has been suggested to serve as an extracellular Na^+^ reservoir in the perinodal space [reviewed in (Poliak and Peles, 2003)].

*Trembler-J* mice have a point mutation in the peripheral myelin protein 22 gene and are a model for CMT type 1A. The sciatic nerves of these mice often showed binary clusters of Nav channels, heminodes (see Figure 3E), and Kv channels aberrantly moved into the paranodal axon membrane in association with improperly formed paranodes (Devaux and Scherer, 2005). Furthermore, the *Trembler-J* heminodes and nodes contain two unusual ion channel subtypes: Nav1.8 and Kv3.1b. Nav1.8 is a tetrodotoxin-resistant Nav channel subtype normally expressed in nociceptive sensory neurons and may have important roles in pain [reviewed in (Eijkelkamp et al., 2012)], but is not usually detected at nodes (Devaux and Scherer, 2005). Kv3.1b is detected at CNS, although usually not PNS, nodes where it may participate in the repolarization of the axon (Devaux et al., 2003). In addition, Nav1.8 was present in demyelinating lesions. In mutant mice deficient in myelin protein zero, nodal, paranodal and juxtaparanodal components were disorganized, and Nav1.8 was abnormally up-regulated at nodes (Ulzheimer et al., 2004), similar to findings in human pathology in CMT type 1B (Bai et al., 2006). CMT type 1C is linked to missense mutations in SIMPLE (small integral membrane protein of lysosome/late endosome). Transgenic mice expressing CMT type 1C-linked human SIMPLE mutant develop a late-onset motor and sensory neuropathy associated with abnormal myelin infolding, paranodal defects, and altered nodal organization (Lee et al., 2013).

Disruption of nodes of Ranvier may also contribute to the pathophysiology of immune-mediated demyelinating neuropathies. In superficial peroneal nerve biopsy from patients with CIDP, paranodal structures were altered, Caspr expression was up-regulated and diffusely localized along the internodal segments (Cifuentes-Diaz et al., 2011). Nav and KCNQ2 nodal channels were less altered but were also detected in the internodes. Intriguingly, a study on the axonal membrane properties suggests that the decreased Nav channel densities at the node may be involved in the conduction slowing in CIDP patients (Cappelen-Smith et al., 2001). In spinal roots from EAN, an animal model for AIDP, nodal Nav channel immunofluorescence changed from a highly focal ring to a more diffuse pattern and, as the disease progressed, eventually it was undetectable (Novakovic et al., 1998).

Chronic pain may in part result from partial demyelination during chronic injury, which creates aberrant Nav channel clusters that may serve as sites of ectopic sensitivity or spontaneous activity [for review see (Levinson et al., 2012)]. Analyses of extracted human dental pulp showed demyelination, altered localization of Nav channels and Caspr clusters, and increased Nav1.7 in the nociceptive primary afferents from painful samples diagnosed with irreversible pulpitis (Luo et al., 2008; Henry et al., 2009). Nav 1.7 is expressed in peripheral sensory neurons, and has been critically linked to pain [reviewed in (Eijkelkamp et al., 2012)]. Similarly, an animal model for neuropathic pain showed disorganized nodes and paranodes (Henry et al., 2006).

There is also evidence that nodes of Ranvier are disrupted in metabolic diseases. The disruption of paranodal axo-glial junctions was found in sural nerve biopsy specimens from patients with polyneuropathy associated with type 1 diabetes mellitus, a disease caused by the autoimmune destruction of pancreatic islet cells and loss of insulin production (Sima et al., 1988). In an animal model for type 1 diabetic neuropathy, nodal and paranodal molecules were displaced laterally, and levels were significantly decreased after eight months of diabetes (Sima et al., 2004). In a separate study on the streptozotocin-induced diabetic rat, immunohistochemistry showed a significant down-regulation of nodal Nav channel signal intensity in sciatic nerves, suggesting that nodal Nav channel protein levels may be reduced with diabetes (Hong and Wiley, 2006). Taken together, these findings both in human pathology and animal models strongly support the idea that altered localization and/or aberrant expression of ion channels at nodes contribute to the pathophysiology in a wide variety of PNS demyelinating diseases.

### Nodal disruption during demyelination in the CNS

Similar to the PNS, myelinating oligodendrocytes play critical roles during formation and maintenance of CNS nodes of Ranvier. For example, in the Shiverer mutant mouse, characterized by a disrupted myelin basic protein gene and loss of myelin basic protein and compact CNS myelin, axo-glial junctions although still present were irregular in shape, size, and distribution (Rosenbluth, 1981). Nav channel clusters were highly irregular and dramatically reduced in number (Rasband et al., 1999). An adult-onset chronic demyelination model (heterozygous transgenic mice with two extra copies of the proteolipid protein gene) exhibited profound reduction in Nav1.6 clusters, loss of the paranodal axo-glial apparatus, and a marked increase in Nav1.2 (Rasband et al., 2003). Thus, as found in many PNS models, abnormalities in CNS myelination affect the molecular organization at and around nodal axons leading to disturbances in CNS functions.

MS is an autoimmune-mediated disease targeting CNS myelin. Disruption of the paranodal architecture and aberrant molecular organization occurs in human MS pathology (Wolswijk and Balesar, 2003; Craner et al., 2004; Howell et al., 2006; Coman et al., 2006). Importantly, the alteration of paranodal molecules and aberrant Kv1.2 localization to paranodal regions may be an early sign of myelin damage preceding disruption of nodes of Ranvier (Howell et al., 2006). Similarly, in relapsing EAE, an animal model for MS, paranodal domain injury precedes formation of internodal demyelinating lesions (Fu et al., 2011). The early disruption of the paranodal axoglial domain may be in part due to microglial activation (Howell et al., 2010). As a likely consequence of nodal and paranodal disruption, a significant switch from Nav1.6 to Nav1.2 expression occurs and both Nav1.2 and Nav1.6 immunoreactivities are diffusely distributed along extensive regions of demyelinated axons within acute MS plaques in human pathology (Craner et al., 2004) and in an EAE (Craner et al., 2003). In addition, recent works suggest that the paranodal and juxtaparanodal axo-glial components could become autoimmune targets (see above) (Mathey et al., 2007; Derfuss et al., 2009). Furthermore, one study points to the presence of perturbed axo-glial interactions in early MS: a large-scale proteomics screen detected multiple molecules that localize to the node and the surrounding axo-glial apparatus membrane in cerebrospinal fluid samples from children during initial presentation of CNS inflammation (Dhaunchak et al., 2012). Thus, the disruptions of neuron–oligodendrocyte interactions and ion channel localization/expression at and near nodes may be one key pathogenic event in MS, especially during the early stage of the disease. For more detailed information about disruption of axon–glial interaction in MS, see (Derfuss et al., 2010; Desmazières et al., 2012).

In addition to MS, traumatic injuries in the CNS may involve disruption of nodes. In an experimental model for spinal cord compression, exposure of juxtaparanodal Kv1.2 channels with accompanying myelin retraction at the nodes contributed to the induction of conduction block (Ouyang et al., 2010). Neonatal hyperoxia exposure in the mouse causes delaying the maturation of the oligodendroglial lineage, myelination abnormalities, axonopathy including paranodal defects and changes in node of Ranvier, and impairment of conduction properties in the adult white matter (Ritter et al., 2013). Also, disruption of the molecular organization of nodes of Ranvier occurs in normal aging. In optic nerves of aged monkey and rat, juxtaparanodal Kv channels were mislocalized to the paranodes and Caspr-labeled paranodes showed irregular profiles, suggesting that age-dependent myelin alterations affect axonal protein localization which is likely to be detrimental to axonal conduction (Hinman et al., 2006). Taken together, these findings underscore the importance of healthy myelinating oligodendrocytes in maintaining the axonal domains at and near nodes, and further demonstrate that nodal disruption may underlie the pathophysiology of various conditions associated with CNS demyelination.

### Nodal disruption caused by neuronal damage

Neuronal damage can also disrupt molecular complexes located on the axolemma at and near nodes causing disturbance of action potential conduction. For example, in chronic idiopathic axonal polyneuropathies, Caspr immunoreactivity at paranodes was irregular, and Nav channels and KCNQ2 channel staining was also increased in internodal regions (Cifuentes-Diaz et al., 2011). The changes in ion channel expression may contribute to the neurological symptoms in ALS (amyotrophic lateral sclerosis), a progressive fatal neurodegenerative disorder characterized by both upper and lower motor neuron losses. In ALS patients, Kv1.2 channel immunoreactivity at the juxtaparanodes was specifically lost in ventral roots (motor), but not in the dorsal roots (sensory), whereas nodal Nav channel expression was unchanged (Shibuya et al., 2011). The loss of Kv channels may cause hyperexcitability of the axonal membrane and lead to the extensive fasciculations (local involuntary muscle contractions), a prominent feature of ALS. In an animal model of traumatic diffuse brain axonal injury produced by fluid percussion insult, calpain-mediated proteolysis of cytoskeletal and scaffolding proteins, ankyrinG at nodes and αII spectrin at paranodes, was associated with nodal damage, suggesting a possible contribution of nodal disruption to the complex mechanisms of traumatic brain injury (Reeves et al., 2010). Finally, the genes encoding ankyrinG (enriched at the AIS and at nodes) and Caspr2 (enriched at the AIS and juxtaparanodes) were identified as major susceptibility loci for bipolar disorder (Ferreira et al., 2008; Leussis et al., 2012) and mental retardation (Zweier et al., 2009), respectively. In post-mortem brains from individuals with bipolar disorder not exposed to antipsychotics, genes encoding ankyrinG, NrCAM, Nav1.6, KCNQ2, and βIV spectrin were significantly altered compared with the brains from bipolar disorder individuals exposed to antipsychotics or controls, suggesting a normalization effect of antipsychotics on the expression of these genes (Chen et al., 2013). Genetic approaches to suppress the mouse gene-encoding ankyrinG in brain, RNA interference and whole brain transgenic knockout, provided evidence for a function of ankyrinG in modulating psychiatric-related behaviors and stress reactivity (Leussis et al., 2013). Furthermore, a recent study suggests the abnormalities in the expression of genes and protein associated with the integrity of the nodes as substrates for the disconnectivity syndrome in schizophrenia (Roussos et al., 2012). The analyses of post-mortem brain samples from patients with schizophrenia revealed significantly reduced mRNA expression of nodal proteins including ankyrinG, and decreased protein levels of ankyrinG in a brain region shown to be vulnerable in schizophrenia. Whether the manifestations of these neuropsychiatric disorders are related to altered functions at nodes and/or AIS is an area that needs further examination.

## CONCLUSION

Nodes of Ranvier are one key structure for proper functioning of mammalian nervous systems. As reviewed here, dysfunction and/or disruption of nodes play significant roles in the development of neurological symptoms. Ion channel functions are disturbed by genetic mutations and by toxins. Autoimmunity against molecules located at and near nodes, myelin defects, and neuronal damage alter the localization and expression of ion channels and disrupt axon–glial interactions. Many important questions still remain to be answered. For example, what is the mechanism of altered expression of ion channel subtypes in the demyelinating lesions? Is it possible to correct the ion channel composition at injured nodes? How do the initial disruption of nodes and paranodal axo-glial apparatus extend to more severe structural damage such as axonal degeneration leading to permanent disability? What is an effective strategy to limit the injury to nodes or to facilitate nodal repair? Research of this nature will continue to uncover the basic mechanisms that underlie the formation, maintenance, disruption, and repair of nodes of Ranvier, and to establish novel therapeutic approaches for currently intractable neurological diseases.
